# A Chimeric HS4-SAR Insulator (IS2) That Prevents Silencing and Enhances Expression of Lentiviral Vectors in Pluripotent Stem Cells

**DOI:** 10.1371/journal.pone.0084268

**Published:** 2014-01-06

**Authors:** Karim Benabdellah, Alejandra Gutierrez-Guerrero, Marién Cobo, Pilar Muñoz, Francisco Martín

**Affiliations:** Human DNA Variability Department, GENYO - Centre for Genomic and Oncological Research (Pfizer/University of Granada/Andalusian Regional Government), PTS Granada, Granada, Spain; Mayo Clinic, United States of America

## Abstract

Chromatin insulators, such as the chicken β-globin locus control region hypersensitive site 4 (HS4), and scaffold/matrix attachment regions (SARs/MARs) have been incorporated separately or in combination into retroviral vectors (RVs) in order to increase transgene expression levels, avoid silencing and reduce expression variability. However, their incorporation into RVs either produces a reduction on titer and/or expression levels or do not have sufficient effect on stem cells. In order to develop an improved insulator we decided to combine SAR elements with HS4 insulators. We designed several synthetic shorter SAR elements containing 4 or 5 MAR/SARs recognition signatures (MRS) and studied their effects on a lentiviral vector (LV) expressing eGFP through the SFFV promoter (SE). A 388 bp SAR element containing 5 MRS, named SAR2, was as efficient or superior to the other SARs analyzed. SAR2 enhanced transgene expression and reduced silencing and variability on human embryonic stem cells (hESCs). We next compared the effect of different HS4-based insulators, the HS4-Core (250 bp), the HS4-Ext (400 bp) and the HS4-650 (650 bp). All HS4 elements reduced silencing and expression variability but they also had a negative effect on transgene expression levels and titer. In general, the HS4-650 element had a better overall effect. Based on these data we developed a chimeric insulator, IS2, combining the SAR2 and the HS4-650. When incorporated into the 3′ LTR of the SE LV, the IS2 element was able to enhance expression, avoid silencing and reduce variability of expression on hESCs. Importantly, these effects were maintained after differentiation of the transduced hESCs toward the hematopoietic linage. Neither the HS4-650 nor the SAR2 elements had these effects. The IS2 element is therefore a novel insulator that confers expression stability and enhances expression of LVs on stem cells.

## Introduction

Retroviruses are efficient vehicles for gene transfer in mammalian cells due to their capacity to stably express a gene of interest in non-dividing and dividing cells. An ideal vector for functional genetics or for gene therapy applications should allow long-term expression of the delivered transgene at physiological levels. Despite the recent success and developments in this area, there are three major concerns that could compromise further applications; insertional mutagenesis [1], epigenetic silencing [2], [3] and variability of expression [4]. These effects are highly dependent on the integration site of the RVs within the chromatin and on the cell type [5].

These considerations are of especial relevance for genetic modification of multipotent stem cells (such as hematopoietic stem cells (HSCs)), as well as for pluripotent stem cells (embroyonic (ESCs) and induced (iPS)). Indeed, the only gene therapy protocols that give rise to leukemia involved first generation γ-RV and HSCs [6], [7]. Highly variable expression and silencing of γ-RV transgenes was the cause of the failure of gene therapy in a clinical trial for X-linked chronic granulomatous disease [8]. Several studies have shown that stem cells can block very efficiently the expression of endogenous as well as exogenous retroviral elements by complex defense mechanisms present in stem cells. Silencing can be mediated through binding of cellular *trans*-acting factors to the proviral long terminal repeats (LTRs) or by methylation of CpG sites within the internal promoter [9], [10], [11]. These effects are highly dependent on the RV vector (γ-RV or lentivirus) and the promoter used (some mammalian and viral promoters have been reported to be resistant to methylation). In particular the use of RVs based on lentivirus (HIV-1), combined with some constitutive promoters (EF1α, PGK, ACTβ) have shown efficient and sustained expression on undifferentiated hESCs [12], [13]. However, differentiation of transduced-hESCs toward different lineages cause silencing of most promoters [14].

Several efforts have been made to deal with transgene silencing and variability of expression. A potential solution is the inclusion of insulators in the viral vectors. Insulators are a complex class of *cis*-acting regulatory sequences that prevent spread of heterochromatin and silencing of genes (barrier activity) and have enhancer-blocking activity [15], [16]. Several insulators have been used on RVs. The chicken hypersensitive site 4 (cHS4) is one of the best characterized and most used [15], [17], [18]. The full length 1.2 kb cHS4 insulator posses both enhancer-blocking (prevent interaction between an enhancer and a promoter) and barrier activity (prevents the spread of condensed chromatin into a transcriptionally active region) [19]. When incorporated into the RVs LTR, the cHS4 insulator provides more uniform gene expression thanks to the enhancer-blocking activity [20]. cHS4 Insulated γ-RVs where able to avoid gene silencing [21] and to decrease genotoxicity by reducing the activation of oncogenes [22], [23]. However, the incorporation of the full-size 1.2 kb cHS4 into the retroviral LTR also causes a drastic reduction in vector titer [24] probably due to the restrictions in reverse transcription and increased homologous recombination [25]. Different studies have shown that the insulator properties of the 1.2 Kb HS4 element are located either in the first 5′ 250 bp (cHS4 Core) [15], the first 5′ 400 pb (cHS4 Extended)[26] and/or in the last 3′ 400 bp [27]. By combining the 5′ Core HS4 (250 bp) with the 3′ 400 bp HS4, Arumugan et al developed a new insulator of 650 bp that had a similar enhancer-blocking and barrier activity effects compared to the full-length 1.2 kb insulator but without effect on vector titer.

As an alternative to cHS4, some authors have included scaffold or matrix attachment regions (SARs/MARs) elements into RVs [28]. SARs/MARs elements bind to the nuclear matrix or scaffold. Within these elements, a consensus bipartite sequence element has been identified and named SAR/MAR recognition signature (MRS) [29]. These MRSs consists of two individual sequences of 8 and 16 bp within a 200 bp distance from each other. DNA sequences that contain MRSs bind with high affinity to the nuclear matrix. However not all SARs contain a MRS, which mean that there are different types of SARs [29]. Insertion of the IFNβ-SAR and IgK-SAR into gammaretroviral (γ-RV) and/or LVs [30], [31], [32], [33] resulted in improved transgene expression probably due to its ability to increase transcriptional initiation rates [34]. SARs/MARs elements are also able to prevent gradual promoter inactivation protecting transgene expression from external epigenetic influences.

However transgene silencing and variability of expression are highly dependent on vector backbone and cell type. Different (sometimes disappointing) results were obtained when the same insulators were used in different vector backbones [35] or when the same insulated vectors were used in different cell types [21], [25], [36]. For example, initial studies found that γ-RVs flanked with the cHS4 only prevented silencing in about 30%–70% of the time depending on the expression cassette [35]. Especially disappointing results were obtained when these insulators were used in human stem cells [25]. Similar contradictory results have been observed when SAR elements were used on different RVs. Ramezani et al showed that the effect of the IFNβ SAR element was variable depending on the vector backbone [32]. They also showed that LVs were better insulated if combining a SAR element with a HS4 insulator (the HS4 located inside the 3′ LTR and the SAR outside the LTR). However the insertion of both elements into the LV backbone reduced the titer to 4×10^5^ TU/ml (10–20 times lower than standard LVs) [32].

Therefore, it would be desirable to design more efficient insulators that maintain or improve enhancer-blocking and barrier activities of previously described elements maintaining vector titer. With this aim, in the present manuscript we designed different insulators and inserted them into a LVs expressing eGFP through the spleen focus-forming virus (SFFV) promoter (SE). The SE LV is highly silenced in hESCs [37] and its expression is highly variable depending on the integration site. The SE/hESCs is therefore a good model to study the different effects of new insulators on LVs. We have constructed an improved insulator, IS2, based on a combination of the HS4-650 and a synthetic SAR element, the SAR2, containing 4 MRS. When included in the 3′LTR of the SE LV, the IS2 insulator was able to enhance expression, avoid silencing and reduce expression variability on human embryonic stem cells during expansion and after differentiation toward the hematopoietic lineage.

## Results

### Construction of LVs incorporating natural and synthetic SAR elements

**Figure 1:**
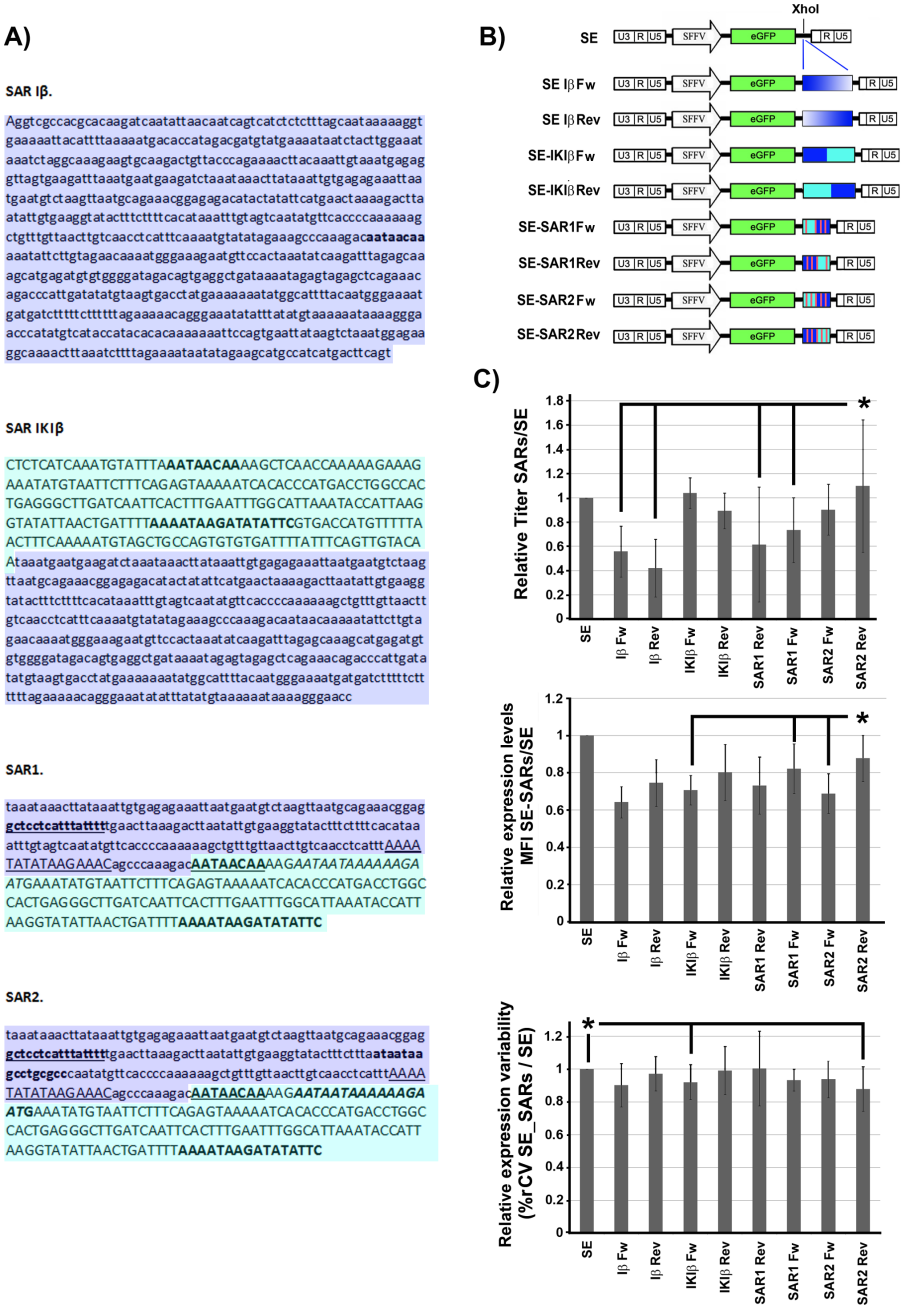
A 388/improved properties than the Interferon β SAR on K562 cells. (**A**) Sequences of the different SARs elements used in the study. SAR Iβ contains 790 bp from the Interferon β locus (AL390882.12: nucleotides 84551–83762). SAR IgKIβ is a chimeric construct containing 240 bp from the Ig Kappa (IgK) consensus SAR sequence (capital case; NG_000834.1: nts 482307–482547) and 480 bp of the SAR Iβ (minor case; AL390882.12: nts 84357–83877). SAR1 and SAR2 synthetic elements are designed to contain 4 and 5 SARs recognition signatures (MRS) respectively. These MRS are embedded into 231 bp of the Iβ locus (AL390882.12; nts 84362–84131, purple) and 157 bp of the IgK locus (NG_000834.1; nts 482316-482473, green). The MRS as described by van Drunen *et al* [29] are: - 1 MRS from the Igk locus (in capital-bold), - 2 MRSs from the β-globin locus (β-globin overlapping: in capital-bold-italic and β-globin +11: in capital-underlined) and - 1 (SAR1) or 2 (SAR2) MRSs from the ψ-globin locus (ψ-globin overlapping: in bold-normal and the ψ-globin +144: in bold-underlined). (**B**) Schematic diagram of the different SE LVs harboring the different SARs. The SARs where inserted between the enhanced green fluorescence protein (eGFP) and the 3′ LTR. (**C**). Effects of the insertion of the different SARs elements on titer (top graph), expression levels (middle graph) and variability of expression (%rCV; bottom graph). All data are represented relative to the parental SE LV. Values represent mean of at least three separate experiments and the error bar indicates the standard error of the mean. (* = p<0.05; two tail unpaired Student *t*-test).

SAR elements are highly conserved non-coding DNA sequences that play an important role in defining independent chromatin domains by their ability to bind to the nuclear matrix. They have been used to improve transgene expression pattern of episomal (plasmids) [38] and integrative (retrovirus) [30], [31] gene transfer vectors. In order to minimize the size of the SAR elements while increasing or maintaining their effect, we designed three synthetic SAR elements (SAR1, SAR2 and SAR IKIβ). SAR IKIβ is a chimera of the consensus Ig kappa (IgK) and interferon β (Iβ) SARs sequences (Figure 1A. See M&M for details). To construct SAR1 and SAR2 elements, 4 or 5 MRSs (from the Ig Kappa (IgK), interferon β (Iβ), β-globin and ψβ globlin loci [29]) were introduced into the Iβ and IgK background (Figure 1A and M&M for details). The different SAR elements were inserted in both orientations into the SE LV using the XhoI restriction site to generate 8 different LVs constructs (Figure 1B).

### The synthetic SAR2 element reduces variation of expression and silencing of the SE LV without affecting viral titer

The SE and the different SE-SARs plasmids containing the different SAR elements (Figure 1B) were used to produce LVs particles as described in M&M. The viral vectors supernatant were used to transduce K562 cells and 7 days later, the transduced cells were analyzed by flow citometry. The percentage of eGFP positive (eGFP^+^) cells were used to determine the transduction units per milliliter (TU/ml) as a measure of viral titer. We detected a small but significant decrease in viral titer when the SE incorporate the SAR Iβ (IβFw; p<0.0001, IβRev; p<0.00001) and SAR1 (p<0.05) in either orientation. However, the incorporation of SAR IKIβ and SAR2 did not affect particle production in either orientation (Figure 1C; top graph).

SARs elements have been described to reduce silencing and variability of expression while increasing transgene expression of RVs[31], [32]. Transgene expression level was assessed by measuring the MFI of the GFP^+^ region. The variation of expression was measured by the robust coefficient of variation (%rCV) of the eGFP^+^ gate obtained using FACSDiva software (see M&M). We couldn't study silencing in these cell line because none of the LVs was silenced in K562 cells (data not shown). The insertion of all SAR elements into the SE LV affected negatively the expression levels (MFI) in this cell line (Figure 1C, middle graph). Still, the negative effect of the SAR2Rev was lower than other SARs elements (p<0.05, comparing SAR2Rev with SAR2Fw, SAR1Fw and SARIKIβFw). Interestingly, we found a significant reduction on%rCV (p<0.05) when the SAR2Rev or the SARIKIβFw were included in the SE LV (Figure 1C, bottom graph).

**Figure 2:**
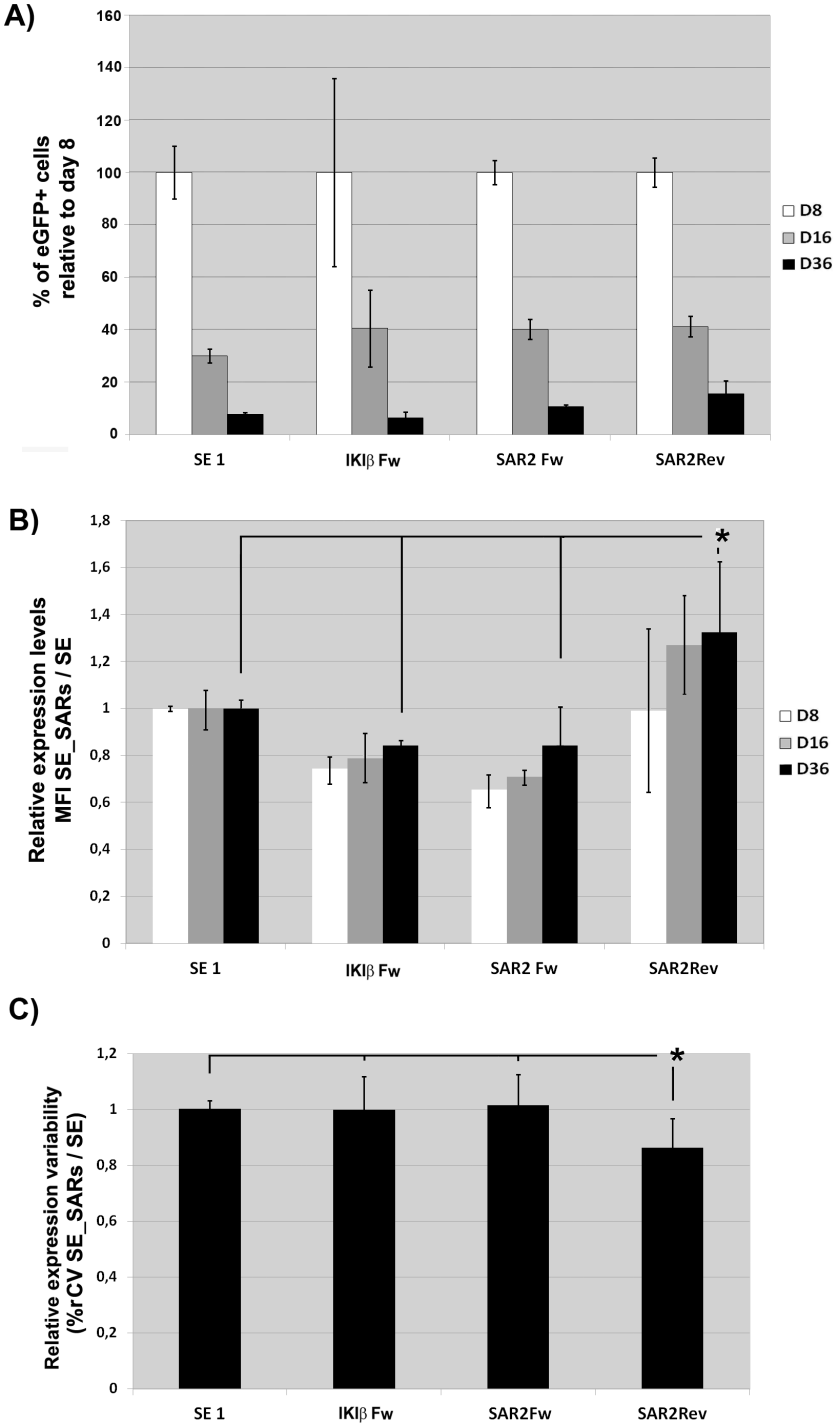
The synthetic SAR2 element improves expression levels and reduces expression variability of the SE LV on hESCs. (**A**). Effect of selected SAR elements on transgene silencing. hESCs were transduced with the SE, SE-IgKIβFw, SE-SAR2Fw and SE-SAR2Rev LVs and the% of eGFP^+^ cells analyzed for 36 days. The data is represented as% of eGFP^+^ cells of each day (days 8, 16 and 36) relative to the% of eGFP^+^ cells at day 8. (**B**) Effect of the selected SAR elements on transgene expression levels. Transduced-hESCs were analyzed at day 8, 16 and 36 post-transduction (D8, D16 and D36) and the data plotted relative to the expression levels of the SE LV. (**C**). Effect of the selected SARs elements on expression variability (%rCV). The graph represents the% rCV (relative to the SE) of the eGFP^+^ cells from cells transduced with the different vectors (see M&M for details). Values represent mean of at least three separate experiments and the error bar indicates the standard deviation of the mean. (* = p<0.05; two tail unpaired Student *t*-test)

Based on its positive effect on expression variability and their better titer compared to the other SARs we selected the SE-SAR2 (Fw and Rev) and SE-SAR-IKIβ (Fw) for further analysis on human embryonic stem cells (hESCs). We first characterized the hESCs in term of pluripotency markers (oct3/4, Tra1-60, Tra1-81, ssea3 and ssea4) to verify that the hESCs maintained their phenotype along culture and after transduction (Figure S1 and data not shown). Contrary to K562, all the LVs analyzed were strongly silenced on hESCs after a short period of time (Figure 2A). Only the SAR2 element in the reverse orientation was able to partially block silencing (although it didn't reach significance; p = 0.06), increase transgene expression levels (Figure 2B; p<0.05) and reduce expression variability (Figure 2C; p<0.05). On the contrary, neither the SE-SAR-IKIβ nor the SE-SAR2FW had a significant effect in any of these aspects. All together our data indicate that the SAR2 element in the reverse orientation is the best choice to improve transgene expression of SE LVs among all the analyzed SARs.

### Evaluation of the effect of different HS4-based insulator on SE LVs

**Figure 3:**
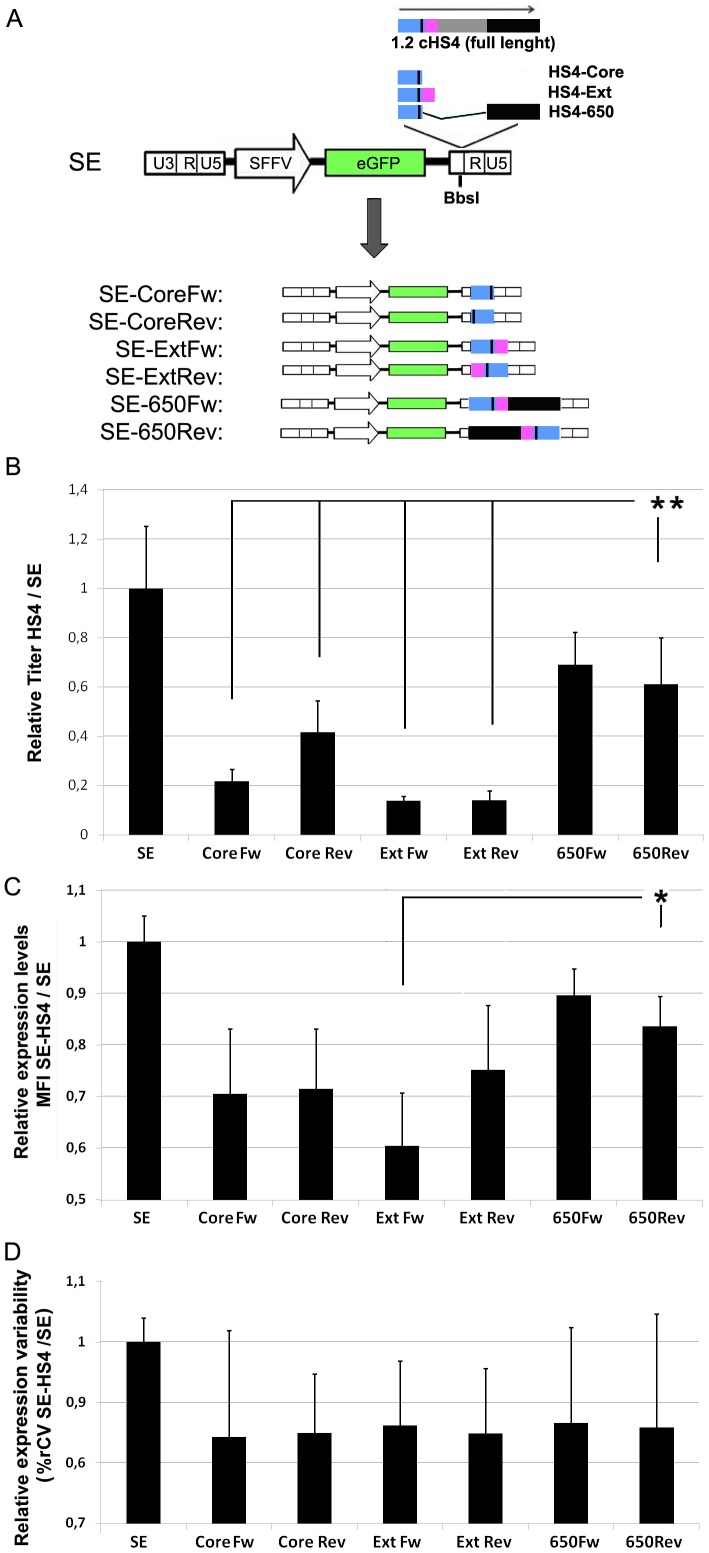
Comparison of the HS4-Core, HS4-Ext and HS4-650 in K562 cells. (**A**). Schematic diagram of the HS4 insulator and the different LV constructed based on the SE LV. The different HS4 elements were inserted into the U3 region of the 3′ LTR (see M&M for details) and the effect of the different HS4 elements on vector titer (**B**), trangene expression (**C**) and expression variability (**D**) compared to the SE LV. (see M&M for details). Values represent mean of at least three separate experiments and the error bar indicates the standard deviation of the mean. (* = p<0.05; two tail unpaired Student *t*-test)

We next analyzed the effect of different HS4-based insulators in SE vectors. The full length 1.2 kb HS4 insulator element is particularly useful when incorporated into RV, reducing the position-effect variegation (variability of expression) and maintaining the transgene expression over time [20]. However its inclusion in RVs can decrease viral titer and transgene expression levels [20], [39]. Different authors have described smaller fragments, 250 bp (Core), 400 bp (Core Extended; Ext) and 650 bp (HS4-650) that maintain the main HS4 characteristics (Figure S2) while minimizing deleterious effects [15], [26], [27]. We synthesised all three HS4 fragments and inserted them into the BbsI site at the 3′LTR of the SE LV in both orientations, forward and reverse (Figure 3A).

We produced all HS4-based LVs together with the original SE and analyzed the viral titer, transgene expression levels, expression variability and silencing on K562 and hESCs. The inclusion of any HS4 insulator into the SE backbone reduced vector titre (Figure 3B) and expression levels (Figure 3C). However, the HS4-650 in either orientation had higher titer than any other HS4-based LV analyzed (Figure 3B; p<0.01 comparing HS4-650 with HS4-Core and HS4-Ext ). In addition SE-650 expression levels were slightly higher than others HS4s, although it was significant only when compared to SE-ExtFw (p<0.05). An important aspect of the HS4 elements is its effect lowering the variability of transgene expression. However, although all HS4 elements reduced slightly the%rCV related to SE, the differences didn't reach significance on K562 cells (Figure 3D).

**Figure 4:**
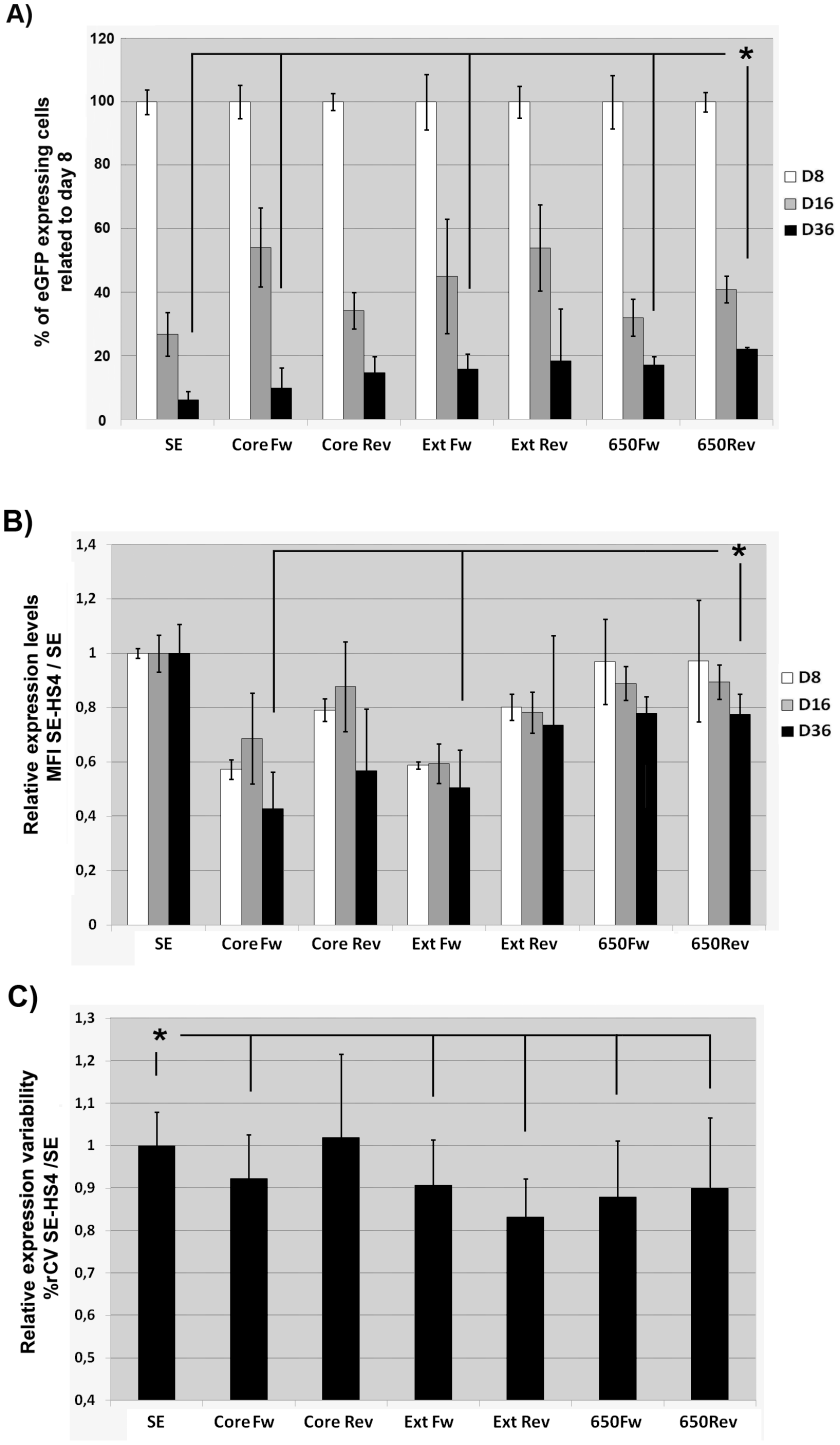
Comparison of the HS4-Core, HS4-Ext and HS4-650 in hESCs. (**A**). Effect of the HS4 insulators on transgene silencing. Graph representing the% of GFP^+^ cells of hESCs transduced with the LVs incorporating the different HS4 insulator in foward (Fw) and reverse (Rev) orientation over time. The data are represented as% of eGFP^+^ cells of each day (days 8, 16 and 36) relative to the% of eGFP^+^ cells at day 8. (**B**). Effect of the HS4 elements on transgene expression levels. Graph representing relative MFI (compared to SE LV) of the eGFP^+^ population from cells transduced with the indicated vectors and analyzed at day 8, 16 and 36 after transduction. (**C**). Effect of HS4 insulators on expression variability. The graph represents the% rCV of the eGFP^+^ cells from cells transduced with the HS4-LV related to the SE LV (see M&M for details). Values represent mean of at least three separate experiments and the error bar indicates the standard deviation of the mean. (* = p<0.05; two tail unpaired Student *t*-test).

The same panel of SE-HS4 LVs was analyzed on hESCs in order to determine the effect of the different HS4s on pluripotent stem cells. We transduced hESCs with SE and with the different LVs incorporating the HS4 elements in the reverse and forward orientation at MOI = 5 and MOI = 1 (to get populations with 40–20% of eGFP^+^ cells). Transduced hESCs were expanded and cultured in undifferentiated state for up to 36 days and analyzed at different time points for% of eGFP^+^ cells (silencing) as well as for MFI (transgene expression levels) and%rCV (expression variability) of the eGFP^+^ population (Figure 4). All elements reduced silencing compared to the original SE LV (Figure 4A; statistics not shown) but the SE-650Rev had the best effect (p<0.0001 compared to the SE LV; p<0.05 compared to SE-CoreFw, SE-ExtFw and the SE-650Fw LVs). As expected, the insertion of HS4 elements also had a negative effect on transgene expression levels in hESCs (Figure 4B; statistics not shown). However, the SE-650 LV presented the highest expression levels of all HS4-based LVs (p<0.05 compared to SE-CoreFw and SE-ExtFw). Finally, our data also showed that the incorporation of the HS4 elements (except the HS4-CoreRev) reduced the variability of expression of the SE vector at similar levels (Figure 4C; p<0.05).

### Improved insulation of LVs by combining SAR2 and HS4-650 elements: The IS2 insulator

**Figure 5:**
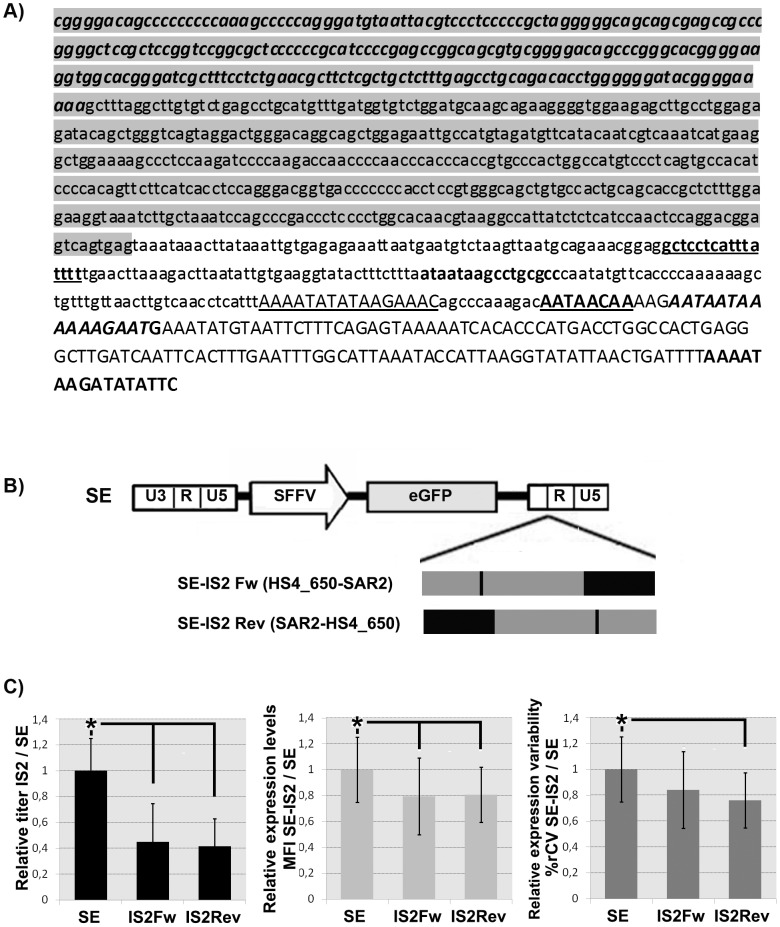
Design of the IS2 insulator based on HS4-650 and SAR2: Effect on K562 cells. (**A**) Sequence of the IS2 synthetic insulator containing the HS4-650 (gray) and the SAR2 element indicating the MRS as in Figure 1A. (**B**) Schematic diagram of the SE viral vector and the IS2 inserted in the U3 region of the 3′ LTR in both orientations. (**C**). Effect of IS2 element on vector titer (left), expression levels (middle) and variability of expression (right) compared to the SE LVs (see M&M for details). Values represent mean of at least three separate experiments and the error bar indicates the standard deviation of the mean. (* = p<0.05; two tail unpaired Student *t*-test).

**Figure 6:**
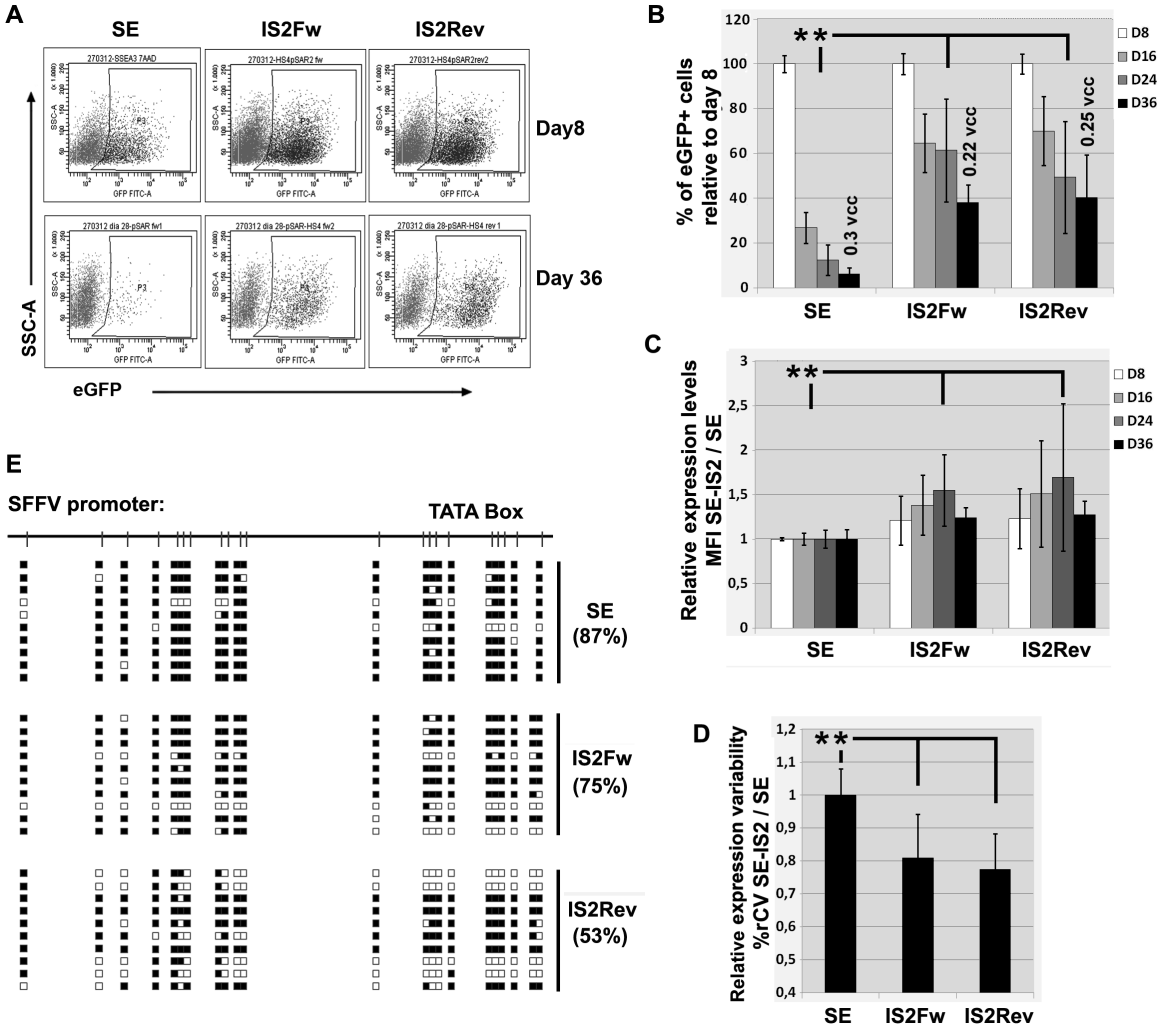
The synthetic IS2 insulator reduces silencing, improve expression levels and reduce expression variability of the SE LV on hESCs. (A) Representative FACS plots of hESCs transduced with the SE, SE-IS2Fw and SE-IS2Rev and analyzed 8 (top plots) and 36 (bottom plots) days post-transduction. (B) Effect of the IS2 on silencing. Graph representing the% of GFP^+^ cells over time (relative to day 8) of hESCs transduced with the SE, SE-IS2Fw and SE-IS2Rev LVs. (C). Effect of the IS2 insulator on expression levels. Graph representing relative MFI (compared to SE LV) of the eGFP^+^ population from cells transduced with the indicated vectors and analyzed at day 8, 16, 24 and 36 after transduction. (D). Effect of the IS2 elements on expression variability. The graph represents the% rCV of the eGFP^+^ cells from cells transduced with the different vectors relative to SE (see M&M for details). (E) SFFV promoter methylation pattern in SE- (top), SE-IS2Fw- (middle) and SE-IS2Rev- (bottom) transduced hESC at day 36 post-transduction. The diagram represents 10 sequenced clones. White and Black Square indicate unmethylated and methylated CpG dinucleotides respectively and the values under the vector names indicate the% of methylated CpGs. Values in B, C and D represent mean of at least three separate experiments and the error bar indicates the standard deviation of the mean. (** = p<0.01; two tail unpaired Student *t*-test).

Based in the results from previous experiments, we selected the HS4-650 and the SAR2 elements to construct a new insulator (IS2). The aim was to create a new insulator that shares the characteristics of both, the SARs and HS4s elements. To construct the new synthetic element, the SAR2 element was located downstream of the HS4-650 (Figure 5A). The IS2 was inserted into the 3′ LTR of SE LV in both orientations (Figure 5B) and its effect on vector titer, transgene expression levels and variability determined on K562 cells (Figure 5C). Both LVs harbouring the IS2 element had about half the titer compared to SE (Figure 5C; left graph), which is a similar reduction on titer compared to what was observed with the HS4-650 insulator (Figure 3B). Similarly, the SE-IS2Fw and SE-IS2Rev also had lower expression levels than the SE LVs on K562 cells (Figure 5C; middle graph; p<0.05). Interestingly, the IS2 insulator inserted in the reverse orientation reduced the variability of expression (Figure 6C; right graph. P<0.05) as was previously observed with the SAR2Rev (Figure 1C; bottom graph). Therefore compared with the SE-HS4s LVs or the SE-SARs, the SE-IS2 had similar titer and better or equal expression levels and variability when studied on K562 cells.

We next analyzed the SE-IS2Fw and SE-IS2Rev LVs on hESCs. We transduced hESCs with IS2-based LVs as well as with the parental SE at MOI = 5 and MOI = 1. Transduced hESCs were expanded in undifferentiated state for up to 36 days and analyzed for% of eGFP^+^ cells, MFI and%rCV by FACS analysis (Figure 6). Both orientations of the IS2 element had a potent effect lowering silencing of the SE LV at all time points (Figure 6A and B. P<0.0001). At day 36 (D36), the SE LVs maintain less than 10% of the eGFP^+^ cells observed at day 8 (D8), while the SE-IS2 kept more than 40% of the D8 eGFP^+^ cells (Figure 6B, black bars). At this time point, SE-transduced hESCs harbored an average of 0.3 vpc while SE-IS2Fw- and SE-IS2Rev-transduced hESCs contained around 0.25 vpc (Figure 6B). These data indicates that the higher percentage of eGFP^+^ cells in the SE-IS2-trasnduced cells reflects lower promoter silencing of IS2-containig vectors compared to the SE. To further corroborate this hypothesis we studied whether the reduction in silencing achieved by the IS2 insulator was due to blocking the novo methylation of the SFFV promoter (barrier activity). We analyzed the methylation status of the SE- and SE-IS2-transduced hESCs at day 36 post-transduction. Bisulphite converted DNA where used as template for nested PCR of the SFFV promoter region encompassing 21 CpG dinucleotides. The PCR products were cloned into the PCR 2.1 vector and individual plasmids sequenced. Figure 6E shows the methylation status (black squares) of the SFFV promoter of 10 clones of the SE, SE-IS2Fw and SE-IS2Rev-transduced hESCs at day 36. Interestingly, the methylation levels correlate with the silencing observed in each sample. Indeed, the SE-transduced hESCs had nearly 90% methylation of the 21 CpG dinucleotides compared to the 50% found in SE-IS2Rev-transduced cells. The differences were even more relevant in CpG sites near the TATA box.

Theoretically, the IS2 element should also improve eGFP expression levels in hESCs due to the presence of the SAR2 (see Figure 2B). As expected, as observed for the SAR2, the SE-IS2Rev and SE-IS2Fw LVs achieved higher eGFP expression levels compared to the SE LV (Figure 6A and C). Importantly, we also observed a strong effect of the IS2 element lowering the expression variability of the SE vector on hESCs (Figure 6D; p<0.01). The enhancer-blocking activity (reduction in%rCV) of the IS2 insulator was superior to the observed for SAR2 and HS4-650 elements (Figures 2C, 4C and Figure S3C; p<0.01).

All together these data showed that the IS2 insulator had stronger barrier and enhancer blocking activities compared to SAR and HS4 elements (Figures 2, 4 and Figure S3) and similar effect than the SAR2 in terms of transgene expression.

### The IS2Rev maintain transgene expression on hESCs after differentiation toward hematopoiesis

**Figure 7:**
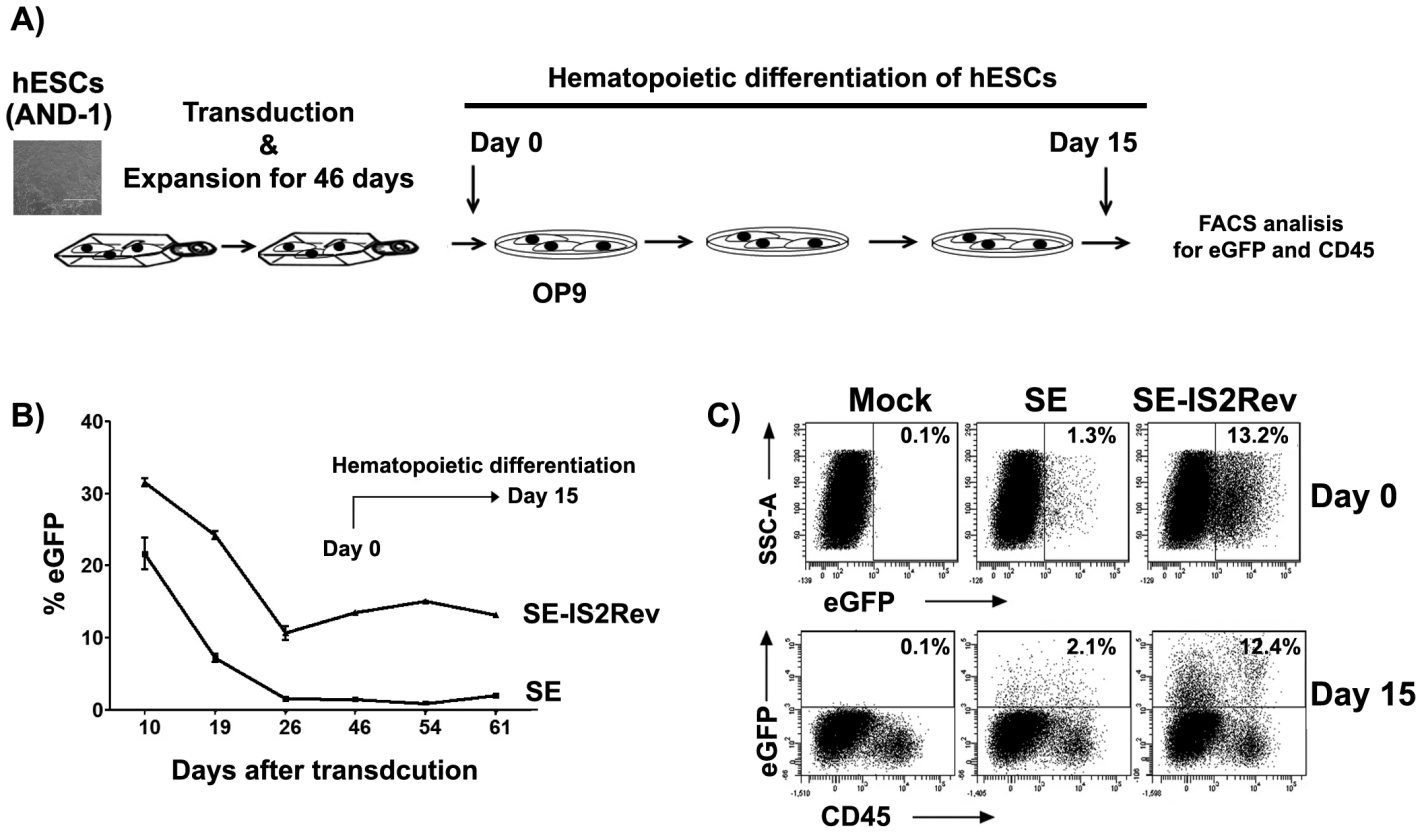
The IS2 insulator protects the SE from silencing during hESCs differentiation toward the hematopoietic lineage. (**A**). Diagram showing the protocol for hematopoietic differentiation of hESC using OP9 stromal cells.(**B**) Graph showing the% of eGFP^+^ cells of SE- and SE-IS2Rev- transduced hESCs at different days during expansion (days 10–46 after transduction) and during hematopoietic differentiation (Days 54 and 61). (**C**) Representative plots showing the percentages of eGFP^+^ cells at day 0 (top panels) and day 15 (bottom panels) after hematopoietic differentiation. The expression of eGFP in CD45^+^ and CD45^-^ cells are showed in the bottom panels.

We finally studied the behavior of the best SE-derived vector, SE-IS2Rev, on hESCs after differentiation toward hematopoietic lineage. We transduced hESCs, maintained the transduced cells in culture for 46 days and them induced differentiation by co-culture with OP9 stromal cells (Figure 7A). We measure eGFP^+^ cells at days 10, 19, 26 and 46 during expansion and at days 8 and 15 after hematopoietic induction (days 54 and 61 post-transduction). Hematopoietic induction was not affected by LV transduction obtaining an average of 12% of CD45^+^ cells at day 5 of differentiation (Figure S4). The IS2Rev prevented silencing during expansion of the hESCs in the undifferentiated stage (as showed previously) and also block further silencing once the hESCs were place onto differentiation (Figure 7B). When analyzed in detailed, we observed that the eGFP^+^ cells were homogenously distributed between the CD45^+^ cells and CD45^−^ (Figure 7C; bottom panels) indicating that the undifferentiated cells (or cells that have differentiated to other lineages) also maintained eGFP expression. In addition, CD45^+^ cells derived from SE-IS2Rev-transduced hESCs had a 3–4 fold increase in eGFP expression levels (MFI) compared to the CD45^+^ cells derived from SE-transduced hESCs (Figure 7C; right-bottom plots and data not shown).

## Discussion

Transgene expression of integrative vectors may be affected by regulatory sequences and heterochromatin flanking the insertion site [2], [3], [4], [5]. Silencing is one of the problems encountered when stem cells are transduced and is mediated by methylation of CpG islands within the integrated provirus promoter [4], [8], [40]. The main objective of the present work is the development of a new insulator that confers more potent enhancer blocking and barrier activities to LVs without affecting viral titer or expression. The effect of insulators on the expression pattern varies depending on the vector backbone and the cell type. We selected a Self-inactivating (SIN) LV backbone expressing eGFP through the Spleen Focus Forming promoter (SFFV) (SE). This LV has been shown to be very stable in some cells lines but to be highly methylated (and therefore silenced) in several stem cell types, including hESCs [37].

To perform our studies we have used K562 cells for vector titration and to see the preliminary effects of the different elements and hESCs as our main target for its relevance on basic research and regenerative medicine. We first performed a systematic comparison of different natural and synthetic SAR elements as well as previously described HS4-based insulators. Based on these data we join the best SAR with the best HS4 to design an improved insulator that combines properties of both elements.

SAR elements play important roles in defining independent chromatin domains by their ability to bind to the nuclear matrix. They have been used to enhance transgene expression and to block de novo methylation of RVs. We designed three synthetic SAR elements and compared with a previously describe SAR element from the IFNβ. The aim was to develop a shorter SAR sequence that, when combined with a HS4 insulator, could fit into a LV without having drastic effects on vector titer. The analysis of LVs harbouring the different SARs showed somehow disappointing results since we could not see any enhancement on transgene expression and the titer was also reduced. This could be due to the strong activity of the SFFV promoter on this cell type that could compete with the SAR elements. However, on hESCs, the shorter SAR2 element (having only half the size of the Iβ SAR) was able to enhance expression and reduce variation of expression. We therefore selected SAR2 as the element for combination with a HS4 insulator.

The HS4 insulator has been extensively studied. The full length 1.2kb HS4 confers two types of insulator activity, enhancer-blocking activity (reduce variation of expression) and barrier activity (preventing proviral silencing). However, the 1.2 kb HS4 has drastic effects on viral titer and transgen expression levels [24], [27]. In order to incorporate the HS4 into RVs, several authors have minimized the size of the HS4 to 250 bp (HS4-Core), 400 bp (HS4-Ext) and 650 bp (HS4-650) which contain the main domains required for its insulator activity [15], [26], [27]. As observed by other authors [15], [26], [27], [32], [39], we observed a reduction in%rCV and silencing but also a general decrease on viral titer and transgene expression levels of all the SE-HS4 LVs. Nonetheless, a lower decrease on vector titer and transgene expression levels was observed for the HS4-650 element in comparison with the HS4-Core and the HS4-Ext. Since the HS4-650 also maintained the barrier activity and the enhancer blocking activity at similar or higher levels than the other HS4, we chose this element for combination with the SAR2 in spite of its larger size.

Some authors showed that the SARs and HS4 elements might only protect viral vector from position effects when used in one orientation [30]. However others reported that the HS4 had insulator properties in both orientations [39]. Our data indicates that the SAR2 element is orientation dependant, reducing%rCV and increasing transgene expression only in the reverse orientation. However HS4 insulator had an overall similar effect independently on the orientation.

Taking all previous results together we combined the best HS4 insulator, HS4-650 (enhancer blocking and barrier activity) with the best SAR element, SAR2 (viral titer, transgene expression and barrier activity) to construct the IS2 element (HS4-650 bp and SAR2). Our initial hypothesis was that this element could share characteristics of the SAR and HS4 elements. In theory the IS2 element could maintain or enhance insulator characteristics (HS4 and SARs) while improving expression (SARs). The insertion of the IS2 element into the SE backbone reduced titer and expression levels on K562 cells. However, these effects were relatively small compared to the drastic effects observed in other insulators [24]. However, on hESCs the SE-IS2 LVs expressed higher eGFP levels compared to the SE LV. The incorporation of the IS2 insulator into the SE LVs in either orientation also conferred potent enhancer blocking (reducing%rCV) and barrier activities (blocking the *novo* methylation and avoiding transgene silencing). However, there were still residual de novo methylations of the SFFV promoter (50%) in the insulated SE vectors (SE-IS2Rev and SE-IS2Fw). This residual methylation correlates with the levels of transgene silencing observed in these vectors after 36 days post-transduction of hESCs. It is therefore possible that the IS2 element is able to block de novo methylation in some chromosomal regions but not in others.

The IS2Rev not only prevented silencing of the SE LV during expansion of undifferentiated hESCs but also during differentiation toward hematopoiesis. Importantly, insertion of the IS2 insulator also increased expression levels of the SE LVs on hESCs in the undifferentiated state as well as after differentiation toward hematopoiesis. This effect on transgene expression was very similar to the increase achieved with the SAR2 element, indicating that the IS2 is able to increase transgene expression levels on hESCs thanks to the presence of the SAR2 sequence.

In summary, we have developed a new insulator, the IS2, based on the combination of a synthetic SAR and the HS4-650 with stronger barrier and enhancer blocking activities that any of the SARs and HS4 elements studied. When inserted in the reverse orientation into the SE LVs, the IS2 was able to prevent silencing, reduce expression variability and enhance expression levels in undifferentiated hESCs and in hESC-derived hematopietic cells. We propose the use of the IS2 element for general applications on LVs that use promoters prompt to silencing on stem cells.

## Materials and Methods

### Cells and reagents

293T Cells (CRL11268; American Type Culture Collection; Rockville, MD) were maintained in Dulbelcco's Modified Eagle's Medium (DMEM, invitrogen, Edinburg, Scotland) supplemented with 10% Fetal Bovine Serum (FBS (Invitrogen), 1% essential amino-acids and antibiotics. The human erythroleukaemic cell line K562 was obtained from ATCC (CCL-243), and maintained in RPMI media (Invitrogen), supplemented with 10% FBS (Invitrogen). AND-1 human embrionic stem cells (hESC) line (provided by the Biobanco del Sistema Sanitario Público de Andalucía. Spanish Stem Cell Bank. www.isciii.es) [41] were maintained undifferentiated in a feeder-free culture, in Matrigel (BD Biosciences, Bedford, MA)-coated p12 plates for lentiviral transduction and expanded in matrigel coated p6 plates. hESC were fed daily with human mesenchymal stem cell-conditioned medium (MSC-CM; Biobanco, Granada. Spain) supplemented with 8 ng/ml βFGF (Miltenyi Biotech, Bergish Glandbach, Germany), as described [42]. Media was changed daily and cells were split weekly by dissociation with 200 U/ml collagenase IV (Invitrogen). Approval from the Spanish National Embryo Ethical Committee was obtained to work with hESCs

### SARs, HS4 and synthetic insulators design and synthesis

The SAR Iβ (790 pb) was obtained from AL390882.12 sequence (nucleotides 84551–83762). Synthetic SAR elements (SAR1: GeneBank accession number KF569214, SAR2: GeneBank accession number KF569215, and SAR IgKIβ: GeneBank accession number KF569216) were constructed by combining different elements. SAR IgKIβ is the result of joining a 240 bp fragment from the Ig Kappa (IgK) locus (NG_000834.1; nts 482307–482547) with a 480 bp from the SAR element of the interferon β (Iβ) locus (AL390882.12; nts 84357–83877). SAR1 and SAR2 contain 4 or 5 M/SAR recognition signatures (MRS) respectively, embedded into a 231 bp fragment of the interferon β locus (AL390882.12; nts 84362-84131) and a 157 bp fragment of the IgK locus (NG_000834.1; nts 482316-482473); The 5 MRSs included belong to the Iβ, IgK, ψβ globlin and β-globin loci as described by van Drunen et al [29]. We constructed three different HS4 based insulators: - the cHS4-Core (a 250-bp fragment from the 5′ of the HS4 element) - the HS4-Ext (a 400-bp fragment containing the cHS4-Core plus 150 bp of the 3′flanking sequences) and - the HS4-650 (a 650 bp fragment which combine distal 3′ 400 bp cHS4 sequences with the 250 bp Core sequence). To construct the IS2 element (GeneBank accession number: KF569217) we combined the HS4-650 and the SAR2.

All sequences were synthesized by Genescript (Genescript USA Inc. NY, U.S.A) and designed to incorporate unique XhoI (SARs) and/or BbsI (HS4s and IS2) restriction sites flanking the insulators.

### SARs and HS4 Lentiviral vector constructs

The SE LV is a self inactivated (SIN) LV expressing the enhanced green fluorescence protein (eGFP) gene under the control of an internal spleen focus-forming virus (SFFV) promoter. The different SARs elements (SAR1, SAR2, IgKIβ and Iβ) were inserted into the unique *XhoI* restriction site of the SE plasmid between the eGFP and the 3′LTR by standard cloning techniques to obtain the SE-SAR1Fw, SE-SAR1Rev, SE-SAR2Fw, SE-SAR2Rev, SE-IgKIβFw, SE-IgKIβRev, SE-IβFw and SE-IβRev. The different HS4-based LV and the IS2-LVs were constructed by first subcloning the LTR fragment into an *XbaI* site of pUC19 (New England Biolabs, Ipswich, MI, USA) to obtain pUC19-LTR. The different HS4 elements (HS4-Core, HS4-Ext and HS4-650) and the IS2 insulator were cloned into the unique *Bbs1* site of the subcloned LTR to generate pUC19-modified-LTRs. The SE-CoreFw, SE-CoreRev, SE-ExtFw, SE-ExtRev, SE-650Fw, SE-650Rev, SE-IS2Fw and SE-ISRev LVs were constructed by removing the XbaI fragment from the SE vector backbone and replacing it with the different X*baI* fragments from the pUC19- modified-LTR. All the constructs generated were verified by automated DNA sequence analysis and with multiple restriction enzyme digestions test.

### Vector production

LVs were produced by transfection of 293T cells with three plasmids: (1) vector plasmid (SE, SE-SAR1Fw, SE-SAR1Rev, SE-SAR2Fw, SE-SAR2Rev, SE-SAR IβFw, SE-SARIβRev, SE-SAR-IgKIβFw, SE-SAR-IgKIβRev, SE-CoreFw, SE-CoreRev, SE-ExtFw, SE-ExtRev, SE-650Fw, SE-650Rev, SE-IS2Fw and SE-IS2Rev); (2) the pCMVΔR8.91 packaging plasmid and (3) VSV-G (pMD2.G) plasmids. (http://www.addgene.org/Didier_Trono). Vector production was performed as previously described [43]. Briefly, 293 T cells were planted on amine-coated petri-dishes (Sarsted,Newton, NC), in order to unsure the 80% of confluence for transfection. The vectors, the packaging pCMVΔR8.9 and envelope plasmid pMD2.G in proportion 3 2 1 were resuspended in 45 µl of LipoD293 (SignaGen, Gainthersburg, MD, USA). The plasmid-LipoD293 mixture was added to pre-washed cells and incubated for 6–8 h. After 48 hours viral supernatants were collected, filtered through 0.45 µm filter (Nalgen, Rochester, NY) and concentrated by ultrafiltration at 2000 g and 4°C, using 100 Kd centrifugal filter devices (Amicon Ultra-15, Millipore, Billerica, MA) or by ultracentrifugation. Concentrated vector were either directly used or frozen at −80°C.

### Vector titration and determination of vector copy per cell

For all the LVs considered in this paper, the vector titration was performed as previously described [44]. Briefly, 100.000 K562 and/or 293 T cells were transduced with serial dilutions of vector supernatant and the percentage of eGFP^+^ cells determined by FACS 7 days later. Once the percentage of eGFP^+^ cells is determined, vector titration is calculated according to the formula: [(10^5^ plated cells ×%GFP^+^ cells) ×1000]/µl of vector.

The quantification of transduction units per milliliter (TU/ml) allows transductions to be conducted based on the number of vector particles added per target cell and will therefore be standardized yielding more reproducible results. The numerical relationship between vector particles used per single target cell is designated as the multiplicity of infection (MOI).

The vector genome per cell of transduced hESCs was calculated using 0.6 µg of genomic DNA ( = 100,000 hESCs) and 10-fold increasing amounts of plasmid DNA (from 10^2^ to 10^7^ copies) for the standard curve. The Q-PCR (Mx3005P, Stratagene, La Jolla, CA) reaction consisted of 40 cycles at 94°C (15 sec), followed by 60°C (30 sec) and 72°C (30 sec). eGFP primers: forward: 59- GTTCATCTGCACCACCGGCAAG- 39 and reverse 59-TTCGGGCATGGCGGACTTGA-39.

### Cells transduction

Freshly collected viral particles were used to transduce K562 and hESC cells. For transduction, K562 cells were washed with Dulbecco's PBS (1×) (PAA Laboratories GmbH, Austria) counted and seed it in a 24 well plates, and incubated for 5 hours with different LVs particles at different MOIs (from MOI = 1 to MOI = 0.1). hESC were dissociated with collagenase type IV, scraped off of the feeder and plated on fresh matrigel coated p24 well plates in the presence of the fresh viral particles at different MOIs (MOI  = 5 And MOI = 1) and 4 µg of Polybrene (1,5-dimethyl-1,5-diazaundecamethylene polymethobromide, hexadimethrine bromide) (SIGMA) per ml. Media was changed after 5 hours.

### Flow cytometry

K562 cells were collected, washed with cold PBS containing 0.1% Sodium azide and analyzed. hESC cells were dissociated with collagenase type IV, half of the well planted on fresh matrigel coated 12 well-plates and the rest resuspended in FACS buffer (PBS, 3% FBS, 2 mM EDTA) for analysis. The cell suspension was filtered through a 70 µm cell strainer (BD Biosciences, Bedford, MA). Live cells were identified by exclusion of the 7-AAD dye. Transduced and/or untransduced cells were stained with the antibodies SEEA-3, SSEA-4, TRA-1-60, TRA-1-81 and OCT3/4 (BD Bioscience) for pluripotency analysis. For hematopoietic differentiation, the hESCs-differentiated population were stained with anti-CD45-APC (Miltenyi Biotech, Bergish Gladbach, Germany). The different samples were analyzed using a FACSCanto II Flow Cytometer (Becton Dickinson, Franklin Lakes, NJ) equipped with the FACSDiva analysis software (BD. Biosciences).

### Transgene silencing

The different cell lines were analyzed every 1–2 weeks for up to 6 weeks after transduction. At each time point, the cell lines were prepared as described previously and analyzed for the percentage of eGFP expressing cells. The different percentages obtained at different days were related to the percentage of the same cells analyzed at time zero (first analysis 8 days post-transduction  = D8). In the absence of toxicity of the transgene expressed, a reduction in the percentage of expressing cells over time indicates silencing of integrated vector by epigenetic mechanisms.

### DNA methylation assay

The promoter methylation state was assessed by bisulfite treatment of Genomic DNA and sequencing of the resulting converted gDNA. The method is based on the selective deamination of cytosine but not 5-methylcytosine by treatment with sodium bisulfite. In the presence of sodium bisulfite, all the unmethylated cytosines are chemically converted to uracil, which is amplified as thymine during PCR. In contrast, the methylated cytosines are not converted. The genomic DNA was isolated from cells using the DNaseasy kit (Qiagen, Crawly, UK). Sodium bisulfite treatment of genomic DNA was performed using the EpiTect bisulfit kit (Qiagene), according to the manufacurer's instruction. The converted gDNA was used for a nested PCR amplification using two primers pairs designed based on converted sequences and amplifying the full length SFFV promoter (containing 24 CpG sites). PCR condition were as follows: First round annealing temperature 55°C, 33 cycle; second round annealing temperature 58°C, 36 cycles. The PCR primer sequence used (SFFV promoter: Forward-1, 5′TAG AAA AAG GGG GGA ATG AAA Reverse-1, 5′AAACAA CTC CTC ACC CTT ACT CAC Forward-2, 5′GGG GGA ATG AAA GAT TTT ATT TG Reverse-2, 5′ACC CTT ACT CAC CAT AAT TTC AAC C. The single PCR product band was purified and cloned into PCR 2.1 (Invitrogene), according to the manufacturer′s instruction, and the plasmid DNAs randomly selected, were sequenced with M13 primers.

### Transgene expression levels (MFI) and coefficient of variation (%rCV)

The transgene expression level achieved by each construct was estimated by the value of the mean fluorescence intensity (MFI) of the eGFP (FITC channel of the FACS) using BD FACSDiva software (BDbiosciences). This MFI was taken from the eGFP^+^ cells from experiments were the eGFP^+^ cells contains one integrated vector per genome (% of eGFP^+^ cells from 5–15%). Therefore, the MFI value is a direct measure of the expression levels achieved by one vector in the cell type analyzed.

The expression levels of the vector can be influenced by enhancers present in the host chromatin, near the integration site. We measure the variability of expression of the different LVs by obtaining the%rCV (rSD/median ×100) of the eGFP^+^ population using BD FACSDiva software (BDbiosciences). We took the same considerations as for the MFI (the% of eGFP^+^ cells should range from 5–15%). A reduction on% rCV due to the presence of an insulator reflects its effect as enhancer blocker.

### Human ESCs hematopoietic differentiation in OP9 coculture system

Hematopoietic differentiation was induced as described by Ji et al [45]. Briefly, the hESCs lines were transferred onto OP9 feeders for 15 days and stained with fluorochrome conjugated monoclonal antibodies anti-mouse CD29-PE (to eliminate OP9) and anti-CD45-APC (Miltenyi Biotech, Bergish Gladbach, Germany). eGFP expression during hematopoietic differentiation was analyzed by FACS at day 0 and 15 of differentiation.

### Statistic analysis

All the statistical analysis was performed with the GraphPad Prism 5 software. We applied the two-tail unpaired Student *t*-test.

## Supporting Information

Figure S1 (**A**) Photograph of a hESC colony during hESCs expansion. (**B**) Dot plots showing the parameters used to identify hESCs. Top panels identify the alive hESCs (7AAD) having the appropriate morphology (SSC-A & FSC-A) and set the region (Is APC, Is FITC and Is PE) for the absence of the expression of the different markers (oct3/4, tra1-60, tra1-81, ssea3, ssea4).
(TIF)

Figure S2 **Sequences of the different HS4 elements used in this manuscript.** The 250 bp HS4-Core (top, purple) is included in all three elements. The HS4-Core Extended (HS4-Ext, 400 bp) contain the 250 bp of the Core (purple) plus an additional 150 bp from the adjacent 3′ region (green). The HS4-650 (650 bp) contains the HS4-Core (purple) plus an additional 400 bp from the distal 3′ of the full length 1.2 kb cHS4 element (orange).
(TIF)

Figure S3 **Comparison of the effect of selected SARs, HS4 and IS2 on transgene expression in hESCs.** (**A**) Effect of the different elements on silencing. Graph representing the% of GFP^+^ cells over time (related to day 8) of hESCs transduced with the SE and the SE incorporating the different elements. (**B**) Effect of the different insulators on the SE expression levels. Graph representing relative MFI (compared to SE LV) of the eGFP^+^ population from cells transduced with the indicated vectors and analyzed at day 8, 16 and 36 after transduction. (**C**) Effect of the IS2 elements on expression variability. The graph represents the%rCV of the eGFP^+^ cells from cells transduced with the different vectors related to the SE (see M&M for details). Values represent mean of at least three separate experiments and the error bar indicates the standard deviation of the mean.
(TIF)

Figure S4 **Lentiviral transduction does not affect hematopoietic differentiation potential of hESC.** Untransduced hESCs (NT), SE- and SE-IS2Rev -transduced hESC were induced towards hematopoiesis. At day 15 of differentiation the cells are dissociated and analyzed for CD45 expression to determine the percentage of cells expressing CD45. Data represent individual representative experiments.
(TIF)
