# Supplementary figures and images for: A Chimeric HS4-SAR Insulator (IS2) That Prevents Silencing and Enhances Expression of Lentiviral Vectors in Pluripotent Stem Cells
Source: PLoS One. 2014 Jan 6;9(1):e84268. doi: 10.1371/journal.pone.0084268 (PMC3882226; doi:10.1371/journal.pone.0084268)

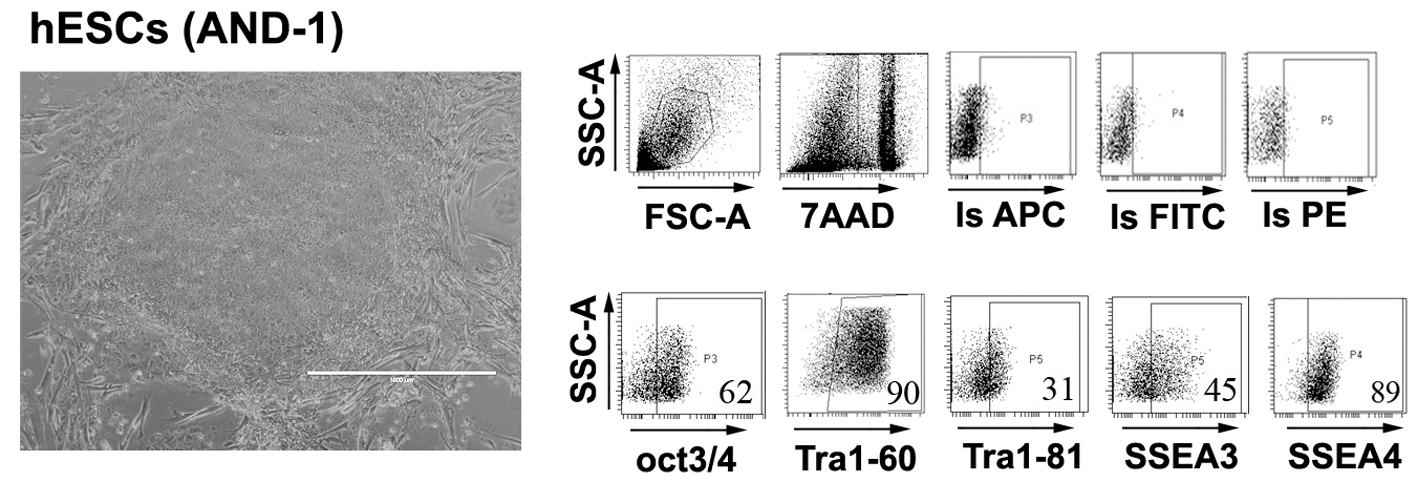

Supplement: Figure S1 — (A) Photograph of a hESC colony during hESCs expansion. (B) Dot plots showing the parameters used to identify hESCs. Top panels identify the alive hESCs (7AAD) having the appropriate morphology (SSC-A & FSC-A) and set the region (Is APC, Is FITC and Is PE) for the absence of the expression of the different markers (oct3/4, tra1-60, tra1-81, ssea3, ssea4). (TIF) [file pone.0084268.s001.tif]

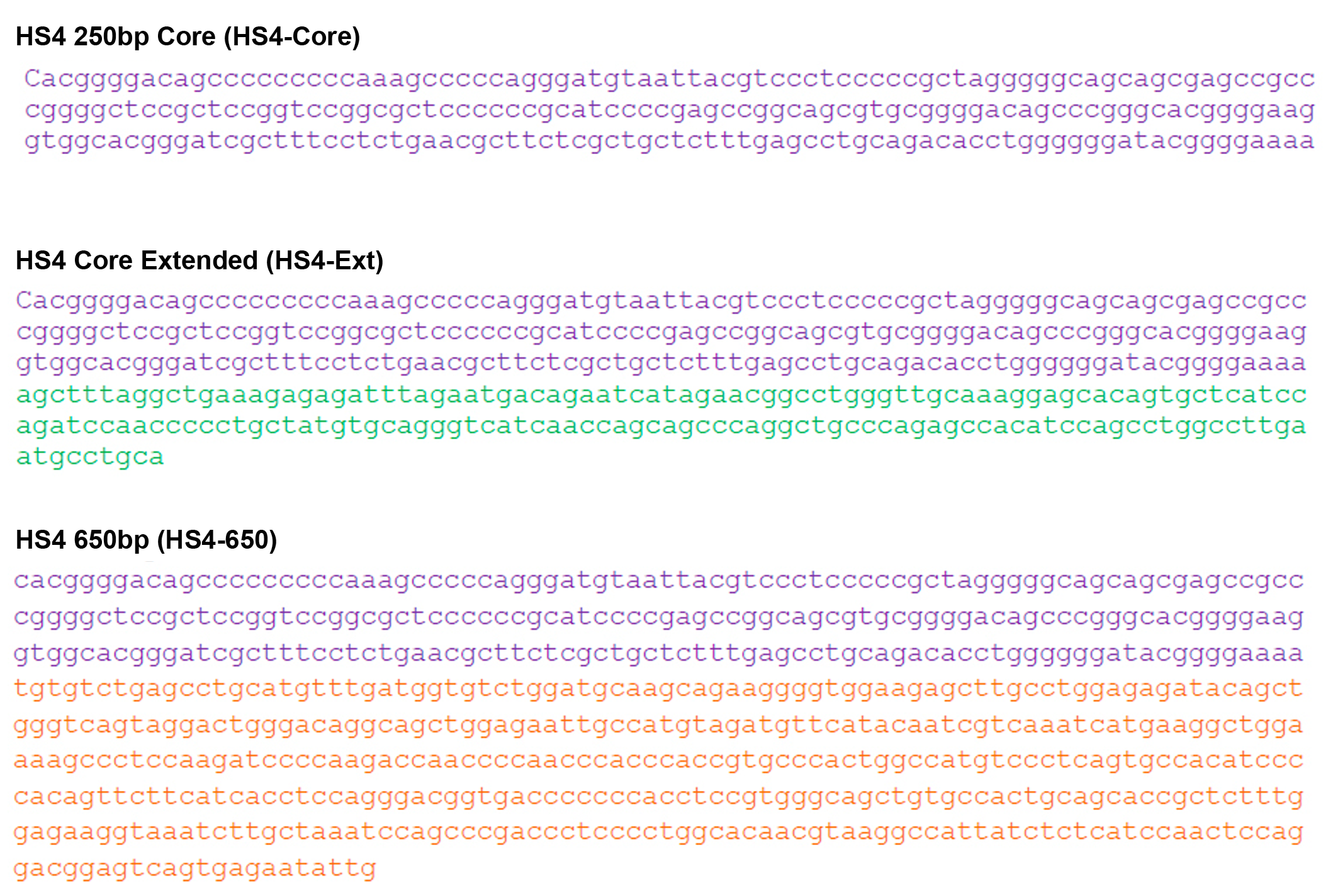

Supplement: Figure S2 — Sequences of the different HS4 elements used in this manuscript. The 250 bp HS4-Core (top, purple) is included in all three elements. The HS4-Core Extended (HS4-Ext, 400 bp) contain the 250 bp of the Core (purple) plus an additional 150 bp from the adjacent 3′ region (green). The HS4-650 (650 bp) contains the HS4-Core (purple) plus an additional 400 bp from the distal 3′ of the full length 1.2 kb cHS4 element (orange). (TIF) [file pone.0084268.s002.tif]

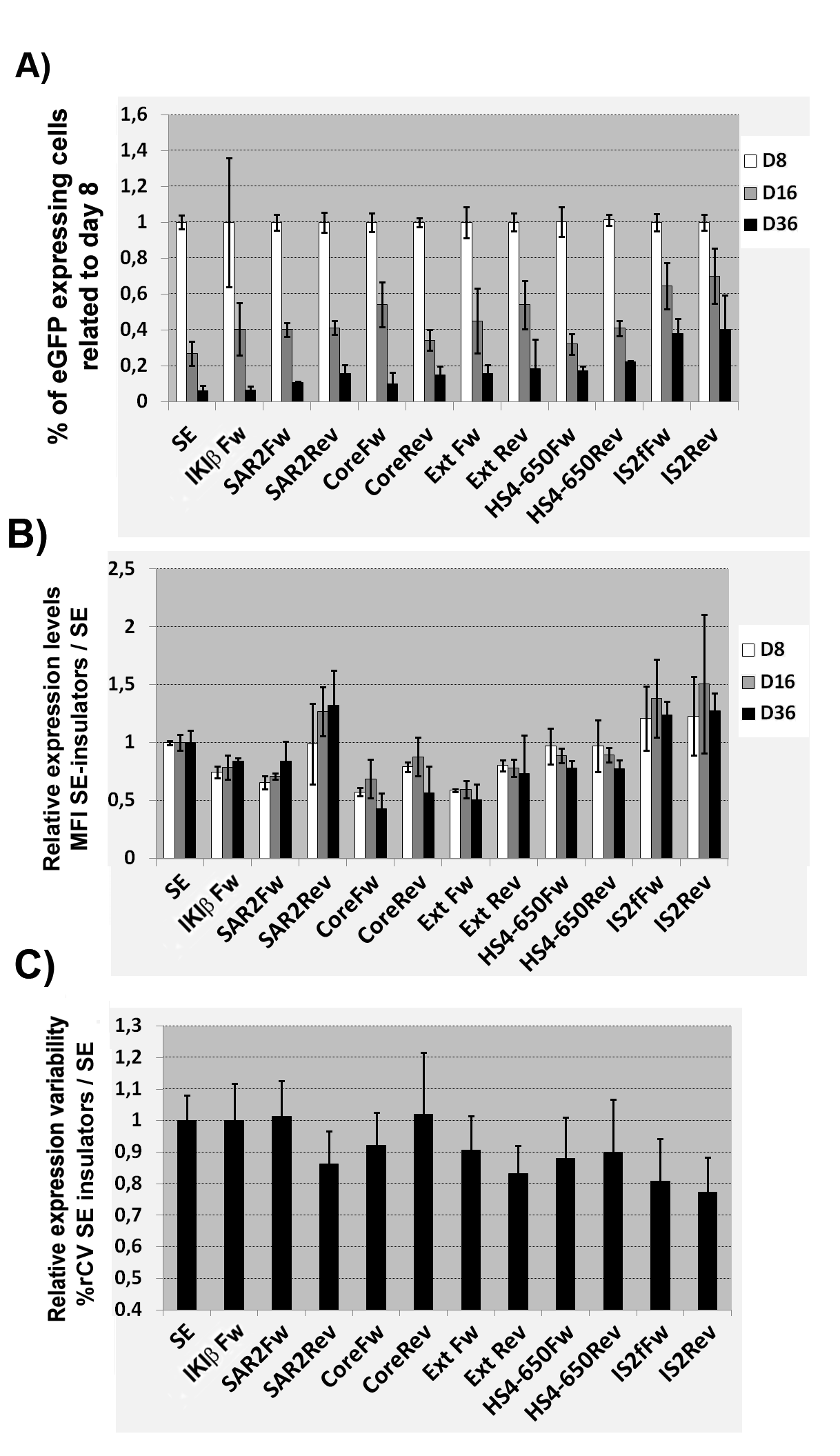

Supplement: Figure S3 — Comparison of the effect of selected SARs, HS4 and IS2 on transgene expression in hESCs. (A) Effect of the different elements on silencing. Graph representing the% of GFP+ cells over time (related to day 8) of hESCs transduced with the SE and the SE incorporating the different elements. (B) Effect of the different insulators on the SE expression levels. Graph representing relative MFI (compared to SE LV) of the eGFP+ population from cells transduced with the indicated vectors and analyzed at day 8, 16 and 36 after transduction. (C) Effect of the IS2 elements on expression variability. The graph represents the%rCV of the eGFP+ cells from cells transduced with the different vectors related to the SE (see M&M for details). Values represent mean of at least three separate experiments and the error bar indicates the standard deviation of the mean. (TIF) [file pone.0084268.s003.tif]

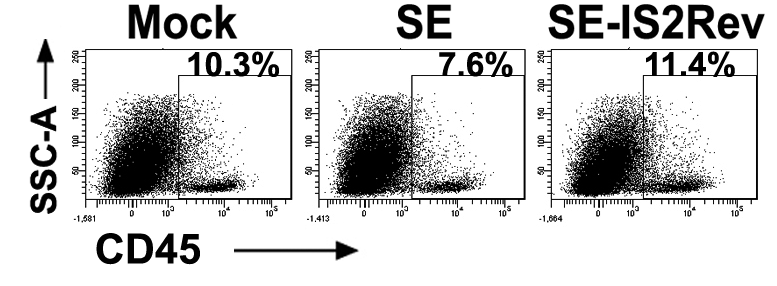

Supplement: Figure S4 — Lentiviral transduction does not affect hematopoietic differentiation potential of hESC. Untransduced hESCs (NT), SE- and SE-IS2Rev -transduced hESC were induced towards hematopoiesis. At day 15 of differentiation the cells are dissociated and analyzed for CD45 expression to determine the percentage of cells expressing CD45. Data represent individual representative experiments. (TIF) [file pone.0084268.s004.tif]
